# Spawning Behaviour and Post-Spawning Migration Patterns of Atlantic Bluefin Tuna (*Thunnus thynnus*) Ascertained from Satellite Archival Tags

**DOI:** 10.1371/journal.pone.0076445

**Published:** 2013-10-01

**Authors:** Guillermo Aranda, Francisco Javier Abascal, José Luis Varela, Antonio Medina

**Affiliations:** 1 Departmento de Biología, Facultad de Ciencias del Mar y Ambientales, Campus de Excelencia Internacional del Mar (CEI·MAR), Universidad de Cádiz, Puerto Real, Cádiz, Spain; 2 Centro Oceanográfico de Canarias, Instituto Español de Oceanografía, Santa Cruz de Tenerife, Spain; Aristotle University of Thessaloniki, Greece

## Abstract

Spawning behaviour of Atlantic bluefin tuna (*Thunnus thynnus*) was investigated using electronic satellite tags deployed in the western Mediterranean spawning ground, around the Balearic Islands (years 2009-2011). All the fish were tagged underwater and released within schools. In general, the fish tagged in the same year/school displayed common migratory trends. Following extended residency around the Balearic Islands, most tagged tuna crossed the Strait of Gibraltar heading for the North Atlantic. Discrepancies between the migratory tracks reconstructed from this and previous electronic tagging studies suggest that the bluefin tuna Mediterranean population may comprise distinct units exhibiting differing migratory behaviours. The diving behaviour varied between oceanic regions throughout the migratory pathways, the shallowest distribution taking place in the spawning ground and the deepest at the Strait of Gibraltar. A unique diving pattern was found on the majority of nights while the fish stayed at the spawning ground; it consisted of frequent and brief oscillatory movements up and down through the mixed layer, resulting in thermal profiles characterized by oscillations about the thermocline. Such a pattern is believed to reflect recent courtship and spawning activity. Reproductive parameters inferred from the analysis of vertical profiles are consistent with those estimated in previous studies based on biological samples.

## Introduction

In spring, Atlantic bluefin tuna, *Thunnus thynnus* (Linnaeus, 1758), perform long seasonal reproductive migrations between feeding areas in the Atlantic Ocean and spawning grounds, either in the Gulf of Mexico (western stock) or the Mediterranean Sea (eastern stock). Like all bluefin tuna stocks, both stocks of the Atlantic bluefin tuna are threatened by overfishing. The continued decline of Atlantic bluefin tuna spawning biomass between the 1970s and 2000s has raised great concern regarding the sustainability of the resource and led to serious questions on the efficacy of current fishery management [1-3], though the most recent assessments show signs of biomass increase, especially in the eastern stock. Ideally, the sustainable management of bluefin tuna stocks should be based on a more comprehensive understanding of the movements and behaviours of the populations over their broad distribution ranges.

The use of electronic archival tags has contributed substantially to the knowledge of the Atlantic bluefin tuna life history, providing valuable data on habitat preferences and migratory patterns. Most electronic tags implanted on Atlantic bluefin tuna have been deployed in the north-western Atlantic Ocean, off the east coasts of the United States of America (US) and Canada [4-17]. On the contrary, electronic tagging experiments conducted in the eastern Atlantic Ocean and Mediterranean Sea are scarce and their results less conclusive [18-27], even though the eastern stock size is around 10 times that of the western population [28-30].

Although major parameters influencing population productivity have been defined in the eastern stock [31,32], essential reproductive features including spawning behaviour and reproductive schedules are still poorly known. Such characteristics are difficult to assess and quantify from conventional field samplings [33], but modern telemetry technologies may help decipher key aspects of the reproductive behaviour in bluefin tunas. For instance, the analysis of movement patterns, diving behaviour and thermal biology based on electronic tagging data has been proposed as a potential tool to identify spawning location and timing in Atlantic bluefin tuna in the Gulf of Mexico [13]. The analysis of pop-up satellite archival tag data have suggested that Atlantic bluefin tuna may use alternative spawning grounds other than those documented thus far [4], a hypothesis that is supported by the finding of larvae outside of the presumed spawning ground in the Gulf of Mexico [34]. Also in the congener species, *T* *. maccoyii* (southern bluefin tuna), a pop-up tag study has revealed more flexible reproductive schedules than previously assumed [35].

The bluefin tuna reproductive season in the Mediterranean Sea extends from May to July. In correlation with a progressive east-to-west increase of the sea surface temperature, the spawning process begins in the Levantine Sea, and then shifts to the southern Tyrrhenian-Malta region and eventually to the Balearic Sea [36]. As in the eastern spawning area, the reproductive season is known to spread over around 3 months (April-June) in the Gulf of Mexico [37]. However, reproductive schedules have not yet been accurately determined at the individual level. Daily spawning has been observed to occur from midnight to sunrise in bluefin tuna breeding schools monitored in the Balearic Sea [38,39], but the proportion of individuals actually engaged in the spawning event, the number of eggs released and the spawning periodicity of each fish in the school are difficult to determine from direct observation of spawning schools.

The purpose of this investigation was to examine horizontal and vertical movements from externally attached pop-up satellite archival tags (PSAT tags) to study the spawning behaviour of Atlantic bluefin tuna in the western Mediterranean Sea. We also analysed the paths and diving behaviours of the tagged tuna throughout different regions of their post-spawning migration to Atlantic habitats. The results of this study might have implications for management and conservation of the species.

## Materials and Methods

Schools of spawning-size Atlantic bluefin tuna were caught by purse-seine during regular commercial fishing around the Balearic Islands early in the spawning season of 2009 (June 14), 2010 (June 8) and 2011 (June 9). Some individuals of the school were implanted underwater with PSAT tags (35 Mk10 and 12 MiniPAT, Wildlife Computers®) as they swam quietly within the purse-seine enclosure, and were immediately released from the enclosure in good condition. These operations involved no harm to the animals and no special permission was required for the development of the experimental activities. Only one Mk10 of the 47 tags deployed failed to transmit, hence this study is based on data from 46 tags (Table 1).

**Table 1:** Details of PSAT tags deployed on Atlantic bluefin tuna in the Balearic area (June, 2009–2011).

Tagging date (dd/mm/yyyy)	Tag ID	Tag type	Tagging position	1st reporting date (dd/mm/yyyy)	1st reporting position	Days at liberty	Total distance travelled (km) (>20 d at liberty)	Time series
14/06/2009	1	Mk10	39.745°N 2.043°E	23/09/2009	48.916°N 27.003°W	101	7,313	No
	2	Mk10	39.745°N 2.043°E	27/06/2009	37.813°N 4.514°E	13	−	No
	3*	Mk10	39.745°N 2.043°E	12/08/2009	44.878°N 8.275°W	59	2,613	No
	4	Mk10	39.745°N 2.043°E	19/06/2009	38.615°N 2.311°E	5	−	No
	5*	Mk10	39.745°N 2.043°E	15/07/2009	38.836°N 0.636°E	31	412	No
	6	Mk10	39.745°N 2.043°E	02/07/2009	38.122°N 1.997°E	18	−	No
	7	Mk10	39.745°N 2.043°E	04/07/2009	38.651°N 4.799°E	20	−	No
	8*	Mk10	39.745°N 2.043°E	20/06/2009	38.653°N 0.651°E	6	−	No
	9*	Mk10	39.745°N 2.043°E	25/06/2009	38.326°N 0.350°E	11	−	No
	10	Mk10	39.745°N 2.043°E	20/06/2009	38.352°N 3.260°E	6	−	No
08/06/2010	11*	miniPAT	38.317°N 1.367°E	14/06/2010	38.341°N 0.145°E	6	−	Yes
	12	miniPAT	38.317°N 1.367°E	23/06/2010	37.699°N 1.769°E	15	−	Yes
	13*	miniPAT	38.317°N 1.367°E	13/06/2010	38.514°N 2.309°E	5	−	Yes
	14	miniPAT	38.317°N 1.367°E	15/06/2010	37.827°N 4.191°E	7	−	Yes
	15	miniPAT	38.317°N 1.367°E	16/07/2010	35.572°N 3.591°W	38	760	Yes
	16*	miniPAT	38.317°N 1.367°E	28/06/2010	38.517°N 0.989°E	20	−	Yes
	17*^,#^	Mk10	38.317°N 1.367°E	07/08/2010	45.016°N 9.193°W	60	2,752	Yes
	18	Mk10	38.317°N 1.367°E	07/07/2010	40.236°N 11.883°E	29	1,099	Yes
	19*^,#^	Mk10	38.317°N 1.367°E	30/08/2010	54.016°N 12.900°W	83	3,732	Yes
	20	Mk10	38.317°N 1.367°E	29/06/2010	38.709°N 2.903°E	21	281	Yes
	21*	Mk10	38.317°N 1.367°E	24/07/2010	35.961°N 5.912°W	46	1,168	No
	22	Mk10	38.317°N 1.367°E	05/07/2010	36.996°N 1.387°E	27	173	No
	23	Mk10	38.317°N 1.367°E	17/07/2010	35.615°N 6.580°W	39	1,039	No
	24	Mk10	38.317°N 1.367°E	25/08/2010	44.369°N 7.923°W	78	3,339	No
	25*	Mk10	38.317°N 1.367°E	30/08/2010	43.722°N 2.520°W	83	3,985	No
	26*	Mk10	38.317°N 1.367°E	08/07/2010	36.459°N 2.054°W	30	514	No
	27	Mk10	38.317°N 1.367°E	23/07/2010	38.003°N 3.486°E	45	488	No
	28*	Mk10	38.317°N 1.367°E	10/06/2010	36.000°N 2.416°E	2	−	Yes
09/06/2011	29*^,#^	miniPAT	38.326°N 0.599°E	19/10/2011	50.738°N 21.888°W	133	6,859	Yes
	30^#^	miniPAT	38.326°N 0.599°E	30/09/2011	63.222°N 14.650°W	114	6,321	Yes
	31^#^	miniPAT	38.326°N 0.599°E	06/11/2011	53.666°N 21.581 °W	151	6,960	Yes
	32	miniPAT	38.326°N 0.599°E	19/06/2011	37.830 °N 0.550°E	11	−	Yes
	33	miniPAT	38.326°N 0.599°E	24/06/2011	38.812 °N 0.426°E	16	−	Yes
	34	miniPAT	38.326°N 0.599°E	12/07/2011	45.895°N 8.310°W	34	2,467	Yes
	35	Mk10	38.326°N 0.599°E	22/06/2011	38.280°N 0.242°E	14	−	Yes
	36	Mk10	38.326°N 0.599°E	06/07/2011	37.661°N 10.451°W	28	1,198	Yes
	37	Mk10	38.326°N 0.599°E	29/06/2011	37.839°N 0.084°W	21	−	Yes
	38*	Mk10	38.326°N 0.599°E	08/07/2011	37.141°N 0.252°W	30	460	Yes
	39*^,#^	Mk10	38.326°N 0.599°E	23/08/2011	51.850°N 11.280°W	76	4,114	Yes
	40	Mk10	38.326°N 0.599°E	29/06/2011	37.004°N 0.526°W	21	323	Yes
	41*	Mk10	38.326°N 0.599°E	09/07/2011	37.705°N 0.032°E	31	451	Yes
	42*	Mk10	38.326°N 0.599°E	30/06/2011	37.807°N 0.386WE	22	343	Yes
	43*	Mk10	38.326°N 0.599°E	20/06/2011	38.349°N 0.374°E	12	−	Yes
	44^#^	Mk10	38.326°N 0.599°E	14/09/2011	48.296°N 8.681°W	98	3,721	Yes
	45	Mk10	38.326°N 0.599°E	28/06/2011	37.987°N 0.075°E	20	−	Yes
	46	Mk10	38.326°N 0.599°E	16/06/2011	38.877°N 0.872°W	8	−	Yes

Estimated horizontal distances travelled are provided only for tags at liberty >20 d. Asterisks on tag code numbers indicate recovered tags. # denotes tags that were used for comparisons of mean depths among areas (see Table 2).

**Table 2:** Mean (means ± SD) and maximum (max) depths recorded in the five regions of the migratory pathways.

		Regions					
	Tag ID	A		B	C	D	E
Day	17	23.5±32.4 (247.5)		43.5±51.4 (232)	61.1±76.1 (370)	−	−
	19	28.2±50.4 (348)		147.1±154.5 (593.5)	69.9±83.1 (384.5)	−	140.6±151.1 (616)
	29	8.4±13.4 (180)>		60.1±75.5 (397)	71.5±91.0 (630.5)	−	73.6±93.0 (616)
	30	14.9±39.9 (572)		40.1±62.9 (458.5)	49.8±88.3 (505)	15.2±31.1 (333.5)	77.7±101.1 (441.5)
	31	45.3±90.6 (615.5)		86.5±100.5 (518)	53.5±104.7 (745)	−	84.2±92.4 (486.5)
	39	16.2±41.2 (369)		57.2±79.8 (358.5)	51.9±77.0 (347.5)	19.5±60.4 (420)	71.6±105.7 (434)
	44	48.7±87.8 (458.5)		57.8±84.4 (458.5)	31.9±54.8 (340.5)	−	24.7±53.9 (237.5)
	Means±SD (max)	29.2±62.3 (615.5)		63.9±91.1 (593.5)	53.9±88.1 (745)	18.3±53.6 (420)	79.9±99.5 (616)
Night	17	17.4±15.4 (178.5)		51.7±59.5 (363.5)	42.8±54.1 (328)	−	−
	19	19.6±31.1 (442)		107.7±197.5 (803.5)	27.6±26.0 (231.5)	−	38.3±66.9 (559.5)
	29	11.6±17. 9 (185)		17.9±35.5 (398.5)	28.5±37.3 (224.5)	−	17.0±28.7 (631)
	30	12.3±19.1 (348)		15.2±39.9 (505)	22.6±44.1 (434)	17.5±24.5 (211)	15.6±31.4 (515)
	31	13.9±19.9 (211)		54.0±92.9 (587)	23.1±27.0 (286.5)	−	15.7±30.4 (615.5)
	39	10.4±15.5 (198)		35.0±73.0 (501)	25.0±23.2 (184.5)	18.2±33.1 (268.5)	25.4±28.1 (326)
	44	15.0±23.5 (326)		37.2±55.5 (214)	24.6±32.5 (230.5)	−	8.9±18.5 (198)
	Means±SD (max)	14.8±21.7 (442)		39.4±88.6 (803.5)	25.4±35.3 (434)	18.0±30.8 (268.5)	17.0±32.1 (631)
Day + night	Means±SD	22.4±49.0		54.8±90.7	42.3±72.0	26.0±64.5	51.7±80.8

Depth values (m) obtained from tags that allowed for time-series data analysis are arranged by day, night and pooled data. (A) Balearic area; (B) Strait of Gibraltar; (C) western Iberian coast; (D) Bay of Biscay; (E) North Atlantic area.

The tags were attached by a monofilament tether to a nylon (umbrella or two-pronged) dart, which was inserted underwater into the dorsal musculature at the base of the second dorsal fin with the aid of a spear gun (Video S1, S2). For identification of the fish after the tag release in case of recapture, the monofilament was wrapped with a conventional spaghetti tag, and then covered externally with silicone tubing. A body mass of 150-200 kg was estimated visually for the tagged fish. Mk10 and miniPAT tags were programmed to record temperature and depth data at 10 and 25 second intervals, respectively, and release 300-360 d after deployment. Tags were programmed to detach in case of fish mortality or premature release, detected as more than 3 d at a constant depth. Once detached from the fish, the tags surfaced and transmitted a summary of the recorded data to the Argos system every 60 seconds over 6-12 d depending on the battery capacity. Datasets downloaded from recovered tags or received through the Argos satellites were processed using the manufacturer software and IGOR Pro 6 (WaveMetrics®). As the tags were deployed on individuals that had already arrived at the spawning ground, the spawning behaviour could not be traced unequivocally from the beginning of the reproductive activity.

Tracks were estimated by CLS using a Kalman filter/smoother approach constrained by light-level, SST and bottom topography data [40]. Some of the tags also transmitted depth and temperature time-series measurements at a 10-min resolution. Mean depths obtained from 7 of these tags (Table 2) were compared among different areas of the migratory pathways using the non-parametric Kruskal-Wallis test followed by the *post hoc* Nemenyi-Dunn test (α = 0.05) [41]. For the assessment of reproductive parameters based on detailed analyses of diving profiles, 13 of the 18 returned tags, which allowed the recovery of full data sets of depth and temperature, were used (Table 3). As described in Results, a characteristic pattern distinguished by high-frequency shallow oscillatory dives (HFSD profiles) was identified in the spawning ground. The person who performed the assessment was unaware of the geolocation data associated with each daily dive profile.

**Table 3:** Assessment of reproductive parameters based on HFSD profiles.

Year	Tag ID	Days spent in the Mediterranean Sea	Days until 1^st^ HFSD profile	Total no. of HFSD profiles	No. of interspersed non- HFSD profiles	Spawning phase duration (d)	Spawning frequency (%)	Spawning periodicity (d)
2009	3	37	2	14	13	27	51.9	1.9
	5	30	0	15	5	20	75.0	1.3
2010	16	−	6	(5)	(1)	−	83.3	1.2
	19	38	1	23	8	31	74.2	1.3
	21	39	11	17	7	24	70.8	1.4
	25	39	8	25	2	27	92.6	1.1
	26	27	1	18	5	23	78.3	1.3
2011	29	−	1	(9)	(0)	−	100.0	1.0
	38	26	1	22	3	25	88.0	1.1
	39	23	3	16	3	19	84.2	1.2
	41	22	3	15	4	19	78.9	1.3
	42	−	3	(10)	(5)	−	66.7	1.5
	43	−	3	(5)	(0)	−	100.0	1.0
Means ± SD		31.22 ± 7.07*	3.31 ± 3.20	18.33 ± 4.00*	5.56 ± 3.40*	23.90 ± 4.11*	80.30 ± 13.44	1.28 ± 0.25

Tags #16, #29, #42 and #43 became detached while still in the Mediterranean Sea, therefore values of“Days spent in the Mediterranean Sea” and “Spawning phase duration” are not available for these tags. Thus, values in parentheses are likely underestimations and were not considered in the calculation of means (asterisks).

## Results

### Horizontal movements

**Figure 1:**
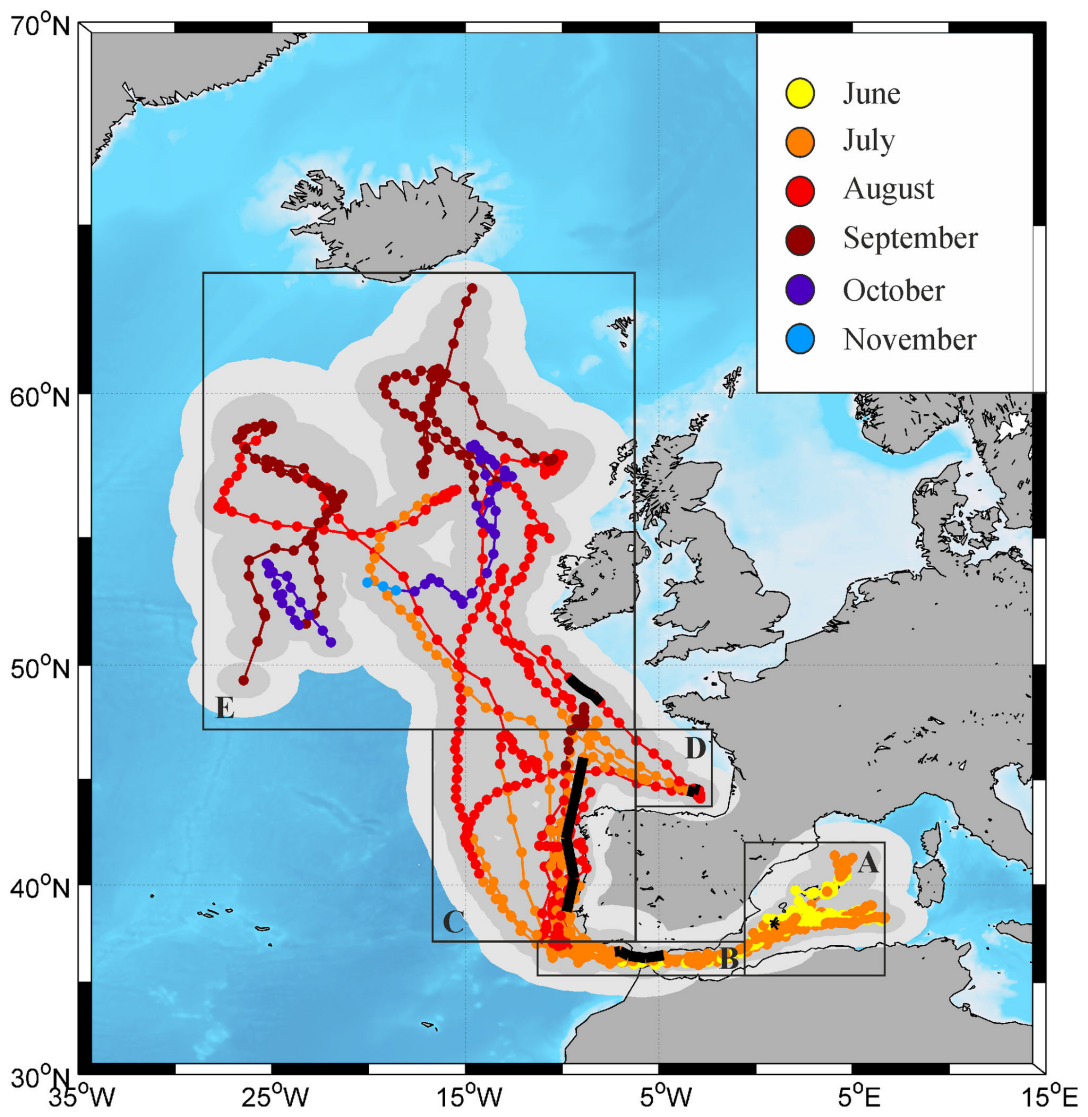
Estimated paths (with 50% and 95% confidence intervals) of 13 Atlantic bluefin tuna tagged in early June, 2009-2011 (≥45 d at liberty). Five successive regions throughout the migratory pathways between the western Mediterranean and the North Atlantic Ocean are distinguished (A-E, black boxes): Balearic area (A), Strait of Gibraltar (B), western Iberian coast (C), Bay of Biscay (D), and North Atlantic area (E). Bold black lines represent five-day coverage of tag #39 track in each of these regions.

Although the tags were programmed to detach 10-12 months after deployment, the maximum retention time was 151 d (miniPAT #31, deployed on June 9, 2011). Overall migratory trends were identified from the paths drawn from the 13 tags that remained attached to the fish for ≥45 days (Figure 1, Table 1). Following a period of residency in the Balearic area, the fish moved in a westward direction, crossed the Strait of Gibraltar and passed through the Gulf of Cádiz, then turned north near Cape St. Vincent and swam fast parallel to the western Iberian coast towards the NE Atlantic. Three fish visited the Bay of Biscay (Cantabrian Sea) before resuming their northward way heading for higher latitudes. The northernmost position recorded was 63.22° N, where tag #30 surfaced off SE Iceland on September 30, 2011 (Table 1). In the following spring (May 26, 2012), the female bearing this tag was captured again at the Balearic spawning ground (~38.20° N 00.50° E), which supports spawning fidelity. The two fish that reached the most westerly estimated positions crossed the 25° W meridian in September, 2009 and 2011, respectively, as they moved southwards after having turned around their northward direction (Figure 1). These fish never neared the 45° W meridian management boundary until their tags came off eventually in the central North Atlantic. The easternmost position recorded was 11.88° E, in the central Tyrrhenian Sea (tag #18, Table 1).

**Figure 2:**
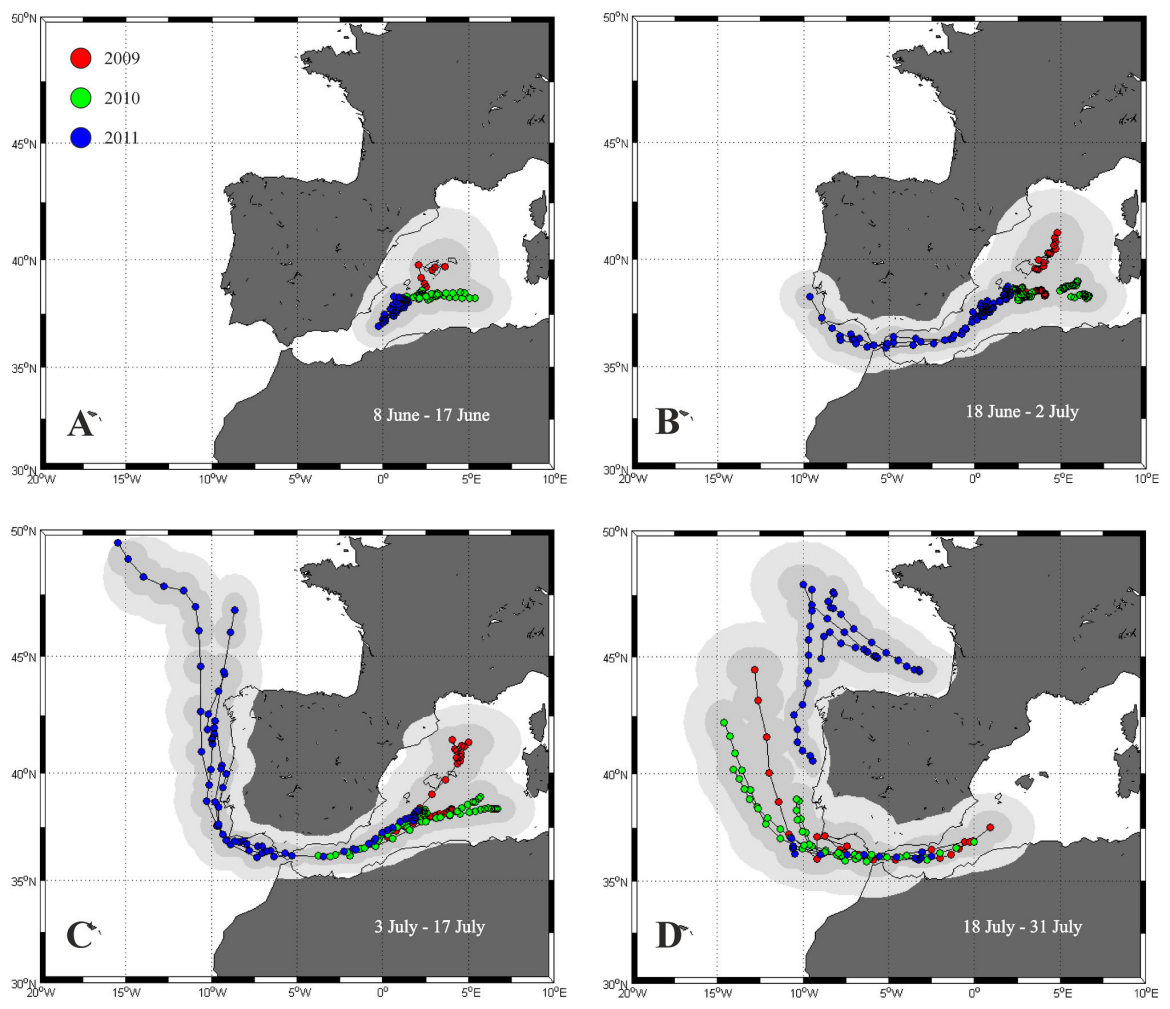
Tracks (with 50% and 95% confidence intervals) from PSAT tags deployed in 2009 (red circles), 2010 (green circles) and 2011 (blue circles). Paths are presented in four consecutive sequences covering the months of June and July. (A) Period between the tagging date (8, 9 or 14 June) and 17 June; this panel includes tracks from 11 tags (2 of 2009, 4 of 2010 and 5 of 2011). (B) 18 June-2 July; 11 tags (2 of 2009, 4 of 2010 and 5 of 2011). (C) 3-17 July; 11 tags (2 of 2009, 4 of 2010 and 5 of 2011). (D) 18-31 July; 10 tags (2 of 2009, 4 of 2010 and 4 of 2011).

A temporal sequence of movements from the spawning ground to the Atlantic Ocean is illustrated in Figure 2. From the deployment date (June 8, 9 or 14) to June 17, all the fish appeared to display a roaming behaviour with non-directed paths, thus suggesting residency in the Balearic Sea associated with reproductive activity (Figure 2A). From June 18 to July 2, the fish tagged in 2009 and 2010 continued to show wandering paths about the spawning ground, while three individuals tagged in 2011 had started the post-spawning migration, either reaching or crossing the Strait of Gibraltar (Figure 2B). From July 3 to 17, the fish tagged in 2009 and 2010 had approached the Strait of Gibraltar, while all but one of the tuna tagged in 2011 had entered the Atlantic Ocean (Figure 2C). From July 18 to 31, none of the tagged fish remained in the spawning area, and most of them were already in the Atlantic Ocean (Figure 2D).

### Vertical movements

**Figure 3:**
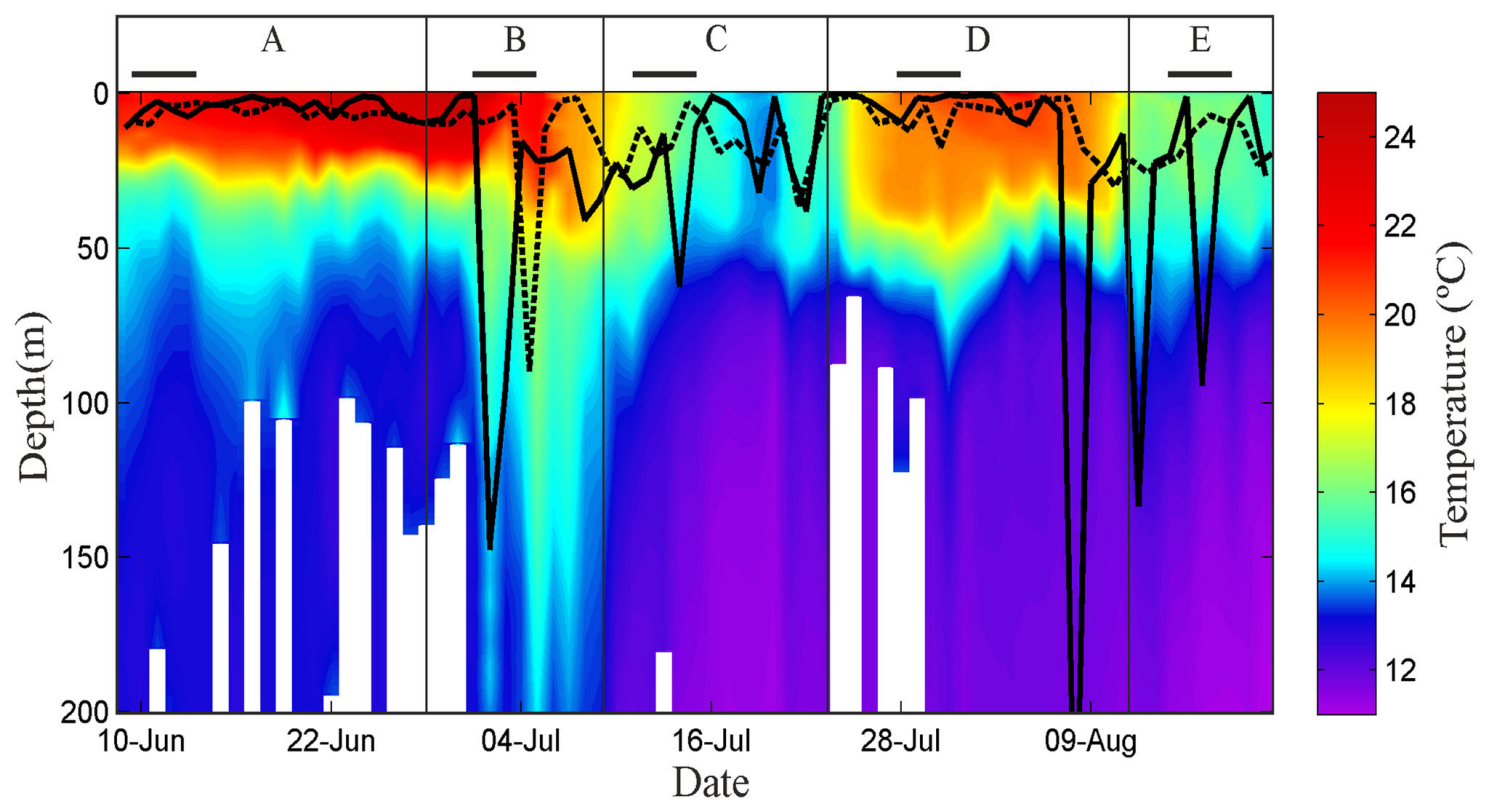
Median depths recorded by tag #39 through regions A-E (see Figure 1). Depths are displayed with the vertical water temperature profile. The water column structure was estimated daily using a locally weighted scatterplot smoothing; then, a contourplot was made with the discrete temperature values (one value per meter and day). Solid line: daytime, dashed line: nighttime. Bars within the box of each region correspond to the respective track segments marked with bold lines in Figure 1.

**Figure 4:**
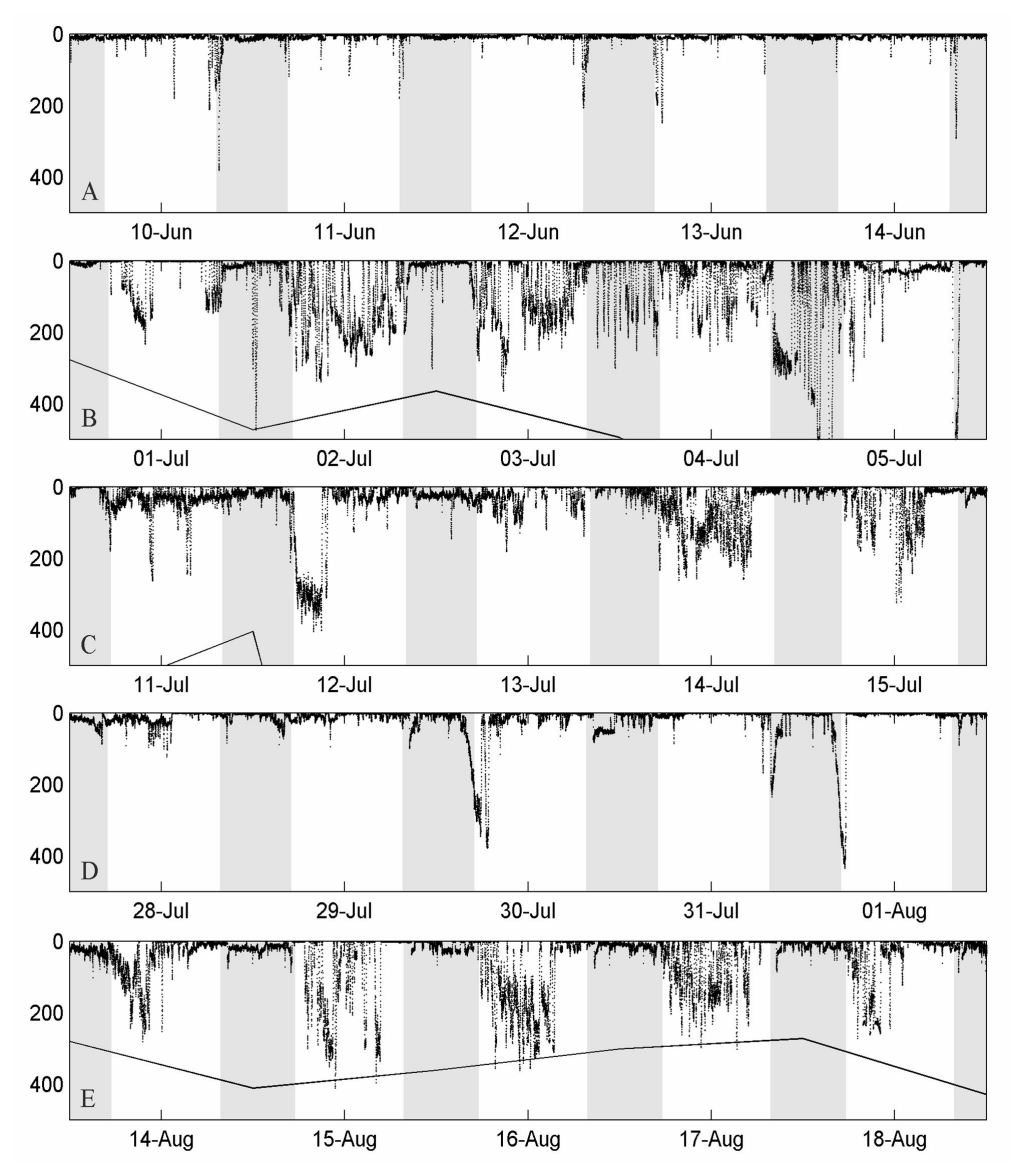
Depth time series of tag #39 through regions A-E in Figure 1. Shaded areas denote nighttime. Solid lines represent the approximate bottom topography.

The diving behaviour between the western Mediterranean spawning ground and the North Atlantic Ocean was analysed from 7 tags capable of generating depth and temperature time-series at 10-min intervals (Table 1) throughout 5 consecutive regions of the migratory route: Balearic area, Strait of Gibraltar, western Iberian coast, Bay of Biscay and North Atlantic area (Figure 1A-E). Plots of median depths and depth profiles from tag #39 (Figures 3 and 4, respectively) exemplify the vertical behaviour of the seven fish throughout the five transited areas. Overall, the median depths observed during night hours were shallower than at daytime, though in the Balearic area and the Bay of Biscay the tagged tuna exhibited shallow diving behaviour all day long (Figure 3A and D).

The mean depths recorded in the five regions (Table 2) differed significantly from each other, both at day and night (Kruskal-Wallis test, *H* = 2008.21, *P* < 0.001, followed by Nemenyi-Dunn *post hock* test, *P* < 0.001). Pooling day and night depth data together, the shallowest diving behaviour corresponded to the Balearic area (22.4 ± 49.0 m, mean ± SD), while the deepest distribution occurred at the Strait of Gibraltar (54.8 ± 90.7 m). The diving pattern in the Balearic area was characterized by shallow daily profiles (29.2 ± 62.3 m) punctuated by spike-dives to ~200 m, occasionally exceeding 350 m (Figure 4A, Table 2) and the shallowest nighttime behaviour of all the studied areas (14.8 ± 21.7 m). In the Strait of Gibraltar area (Figure 4B, Table 2), the mean daytime distribution was deep (63.9 ± 91.1 m), and during the night the fish showed the deepest behaviour observed throughout the post-spawning migratory route (39.4 ± 88.6 m). The maximum depth recorded was reached by an individual (tag #19) that swam for six hours at ~800 m, which is the bathymetric limit in this region. Throughout the stretch alongside the western Iberian coast, the diving behaviour was deeper during the day (53.9 ± 88.1 m), with descents down to ~300 m that resulted in frequent V-shaped profiles and some U-shaped profiles (Figure 4C). A mean depth of 25.4 ± 35.3 m was recorded in this zone at nighttime. In the Bay of Biscay, the tags recorded the shallowest daytime behaviour of all regions (18.3 ± 53.6 m), with occasional U-shaped profiles up to 400 m deep (Figure 4D). During the night, the fish continued to exhibit a shallow distribution (18.0 ± 30.8 m), making brief dives up to 200 m (Figure 4D). In the North Atlantic area (Figure 4E, Table 2), the fish experienced the deepest behaviour during the day (79.9 ± 99.5 m), showing frequent U-shaped profiles up to 300-400 m, whereas they displayed a shallow behaviour at nighttime (17.0 ± 32.1 m).

**Figure 5:**
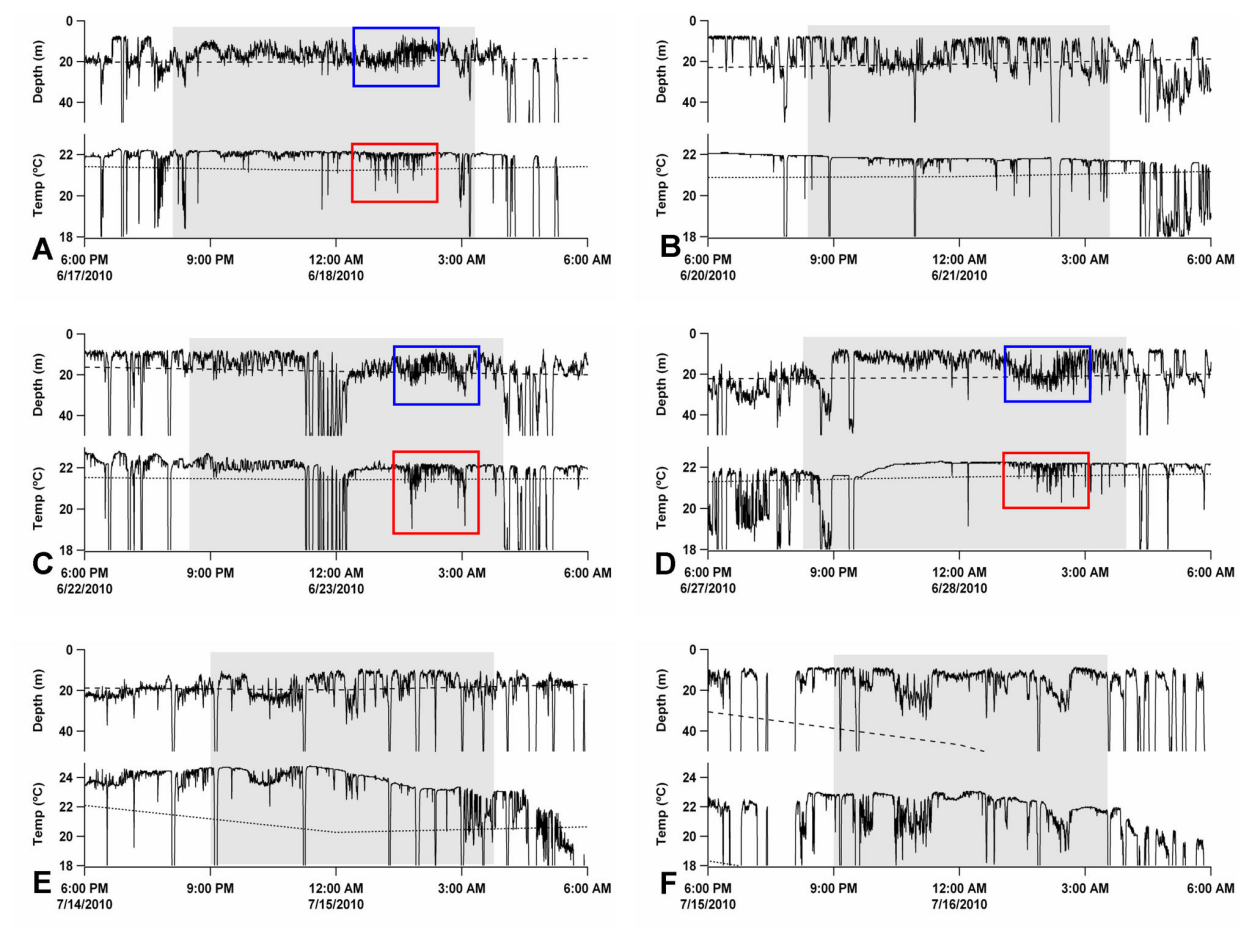
Depth and water temperature time series from tag #25 in the Balearic area. High-frequency shallow dives (HFSD profiles) were detected between midnight and sunrise (A, C, D); this pattern consisted of frequently repeated shallow dives (blue squares) below the bottom limit of the mixed layer (dashed line), which resulted in thermal oscillations (red squares) about the thermocline (dotted lines). Vertical profiles displaying deeper scattered dives and no clear oscillatory pattern (non-HFSD profiles) alternated with daily series of consecutive HFSD profiles (B); this pattern occurred several successive days following the last HFSD profile (E, F).

**Figure 6:**
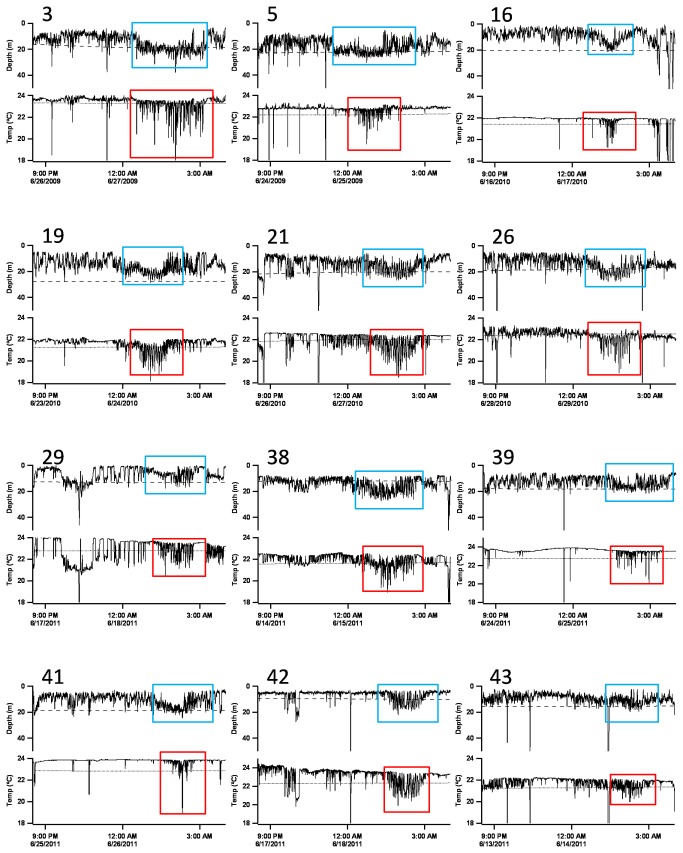
Examples of high-frequency shallow diving behaviour (HFSD) from 12 fish on their spawning grounds. Each panel shows one night of depth and water temperature time series from each tag used for the analysis of vertical movement patterns (see Table 3), except for Tag #25, which is shown in Figure 5. Tag ID is indicated at the top left of each panel.

A distinctive diving behaviour was found on most days while the fish remained in the Balearic area, taking place between midnight and sunrise, and was not repeated elsewhere in any of the migratory stages spanned by this study. Such a pattern was characterized by what we refer to here as high-frequency shallow dives (HFSD). The HFSD profiles were distinguished by permanency at depths of less than 40 m and brief oscillatory movements across the bottom limit of the mixed layer (Figures 5A, C, D and 6, blue squares). Such short dives occurred with a frequency of 2 times every 5 min approximately, and lasted about 90 min on average (range: 45-160 min). These vertical movements resulted in distinctive thermal profiles characterized by densely packed temperature oscillations (Figures 5A, C, D and 6, red squares) that might drop between 2-3 °C. As the spawning season progressed and SST rose to 24 °C, the fish exhibited a slightly deeper distribution below the mixed layer, but they continued to experience the same oscillatory pattern of thermal variations.

HFSD behaviour was not present every single day, but there were nights when the vertical profiles showed no clearly defined pattern, comprising scattered dives that sometimes reached depths over 100 m. On average, such non-HFSD profiles occurred every 4.5 ± 3.2 days of consecutive HFSD patterns (Figure 5B), and also occurred on the subsequent days following the last HFSD profile (Figure 5E and F). The last HFSD occurrence thus could mark the onset of the post-spawning migration.

For the detailed study of vertical behaviours, temperature and depth profiles were constructed using the data downloaded from recovered tags (Table 3). One of these tags recorded HFSD behaviour as early as the first night following the deployment of tags. Four individuals exhibited HFSD patterns on the second night, whereas in the remaining fish HFSD profiles were first observed between 2 and 11 d after the tag deployment (Table 3). The mean elapsed time between the tagging date and the appearance of the first HFSD profile was 3.3 ± 3.7 d. The mean number of HFSD profiles identified throughout the entire spawning phase was 18.3 (range: 14-25) (Table 3). The end of the individual spawning period was established at the day from which HFSD profiles were no longer observed. Therefore, the spawning phase duration was estimated as the time elapsed between the first and last HFSD profiles recorded (i.e., the sum of HFSD profiles plus intervening non-HFSD profiles). Thus, the longest spawning period recorded was 31 d and the shortest 19 d (mean 23.9 d). For the estimation of these values, only the tags that popped off outside of the Mediterranean Sea were considered, in order to make sure that all the fish had completed their reproductive function. The mean ratio between the number of HFSD profiles and the spawning phase duration (i.e., the spawning frequency) was 0.83 d^-1^, whereas the inverse of this value (i.e., the mean spawning periodicity or interspawning interval) was 1.28 d (Table 3).

## Discussion

Studies based on gonad histology and ichthyoplankton surveys have shown that Atlantic bluefin tuna utilize waters around the Balearic Islands to spawn [31,42]. With a view to inferring reproductive behaviour patterns in this region, PSAT tags were affixed on spawning-size fish in the western Mediterranean Sea. The tags provided useful data not only through satellite transmissions but also from downloads of 18 returned tags.

The PSAT tagging procedure involves an issue of trade off between the attachment strength (which is proportional to the expected time at liberty of the tag) and the stress on the fish. The trauma and stress associated with capture and handling may influence the post-release behaviour of some fish species [43]. As the limited useful life of PSAT tags would not allow us to monitor more than one breeding season, we decided to tag the fish underwater in order to reduce the stress level to a minimum, thus preserving their natural behaviour as much as possible. In addition, the schooling tendency of bluefin tuna might cause the tagged individuals to quickly resume normal group behaviour, thus minimizing the impact of the tagging procedure [43]. In contrast, a major disadvantage of underwater tagging by spear gun would be the risk of a deficient insertion of the anchoring system, thus shortening the tag retention time. However, tagging animals on deck rather than in the water does not necessarily appear to increase PSAT retention times [44].

Our geolocation estimates showed that all the tagged fish stayed in the Balearic spawning ground throughout the putative spawning period, with the exception of a single individual, which travelled eastward to the Tyrrhenian Sea. The mean time spent in the spawning area following the tag deployment was about 25 d, and during that time the fish did not exhibit directed movement paths. Although 13 of the deployed tags popped off early (<15 d) in the Mediterranean Sea, those that remained attached for more than 45 d surfaced in the Atlantic Ocean. This suggests that most fish tagged during the reproductive season in the Balearic spawning ground tend to undertake backward migration to Atlantic waters after spawning, passing through the Strait of Gibraltar from late June to late July. Such observations disagree with previous studies showing that many tuna tagged in the Mediterranean Sea did not migrate to the Atlantic Ocean, hence suggesting that some individuals extend their residency time in the Mediterranean for several months or the entire year [19,20,22,24,26,27]. The differences between the present results and earlier reports may be partly explained by the different dates when the fish were tagged. Although the existence of different bluefin tuna subpopulations in the Mediterranean Sea is difficult to detect genetically [45], a recent study [46] concluded that the Mediterranean bluefin tuna population may comprise distinct reproductive units. Some population subdivisions spawning in the central-eastern Mediterranean Sea and exhibiting resident behaviour would intermingle with more mobile bluefin tuna spawning in the central-western Mediterranean. It would then be plausible that highly migrant subpopulations predominate over more resident ones in the westernmost Mediterranean area. According to this hypothesis, tagging surveys carried out at separate times in the western Mediterranean might target different reproductive units with differential migration patterns. Obviously, as the tag deployment date shifted away from the spawning season, the likelihood of tracking movements into the Atlantic Ocean would decrease [25]. This would support the view that the Atlantic bluefin tuna population spatial dynamics is far more complex than generally believed [8,16,28,29]. It would be, therefore, important to extend electronic tagging surveys to the other Mediterranean spawning grounds in order to analyse the dynamics of the eastern population and investigate the potential existence of discrete subunits differing in migratory and reproductive behaviours.

While a previous tagging survey did not find synchronous spatial and temporal patterns in Atlantic bluefin tuna tagged and released singly on a foraging ground [16], we did track synchronous post-spawning movements among tuna tagged within the same school. An overall analysis of the spawning behaviour and the exit timing and trajectories from the western Mediterranean spawning ground indicates that individuals of a school tend to take common post-spawning migratory pathways and possibly share similar reproductive schedules. Schooling behaviour and synchronicity have also been proved in yellowfin tuna by acoustic tracking [47].

This is the first report describing vertical movements of Atlantic bluefin tuna breeders tagged on a Mediterranean spawning ground. In addition to horizontal tracking, thorough analyses of vertical movements have proved extremely useful in determining behavioural patterns and habitat utilization of tunas [10,13-15,17,48-52]. The vertical behaviour of the PSAT-tagged tuna varied significantly at different stages of their post-spawning migratory pathways. Three distinct daily vertical movement patterns have been distinguished in Atlantic bluefin tuna [15] that fit well with our observations. A restricted profile, characterized by prevalent extended periods of swimming in surface waters and occasional bounces, was found in the Balearic area and the Bay of Biscay. V-shaped profiles, which are thought to represent transiting or searching behaviour, were observed in East Atlantic migration stretches parallel to the western Iberian coast and in open waters. U-shaped profiles, which are believed to be associated with feeding behaviour in the deep scattering layer, occurred in the Bay of Biscay and, more significantly, the North Atlantic area.

The deepest dives were recorded as the fish passed through the Strait of Gibraltar entering the Atlantic Ocean. Interestingly, a similar deep diving behaviour was observed in Atlantic bluefin tuna entering and leaving the Gulf of Mexico spawning grounds [10,13], and during the passage across the Strait of Gibraltar in the reproductive migration to the Mediterranean Sea [15]. It has been argued that deep dives performed near the Strait of Gibraltar allow the fish to locate Mediterranean outflow water and thus function to guide them into the Mediterranean [15]. In the Strait of Gibraltar, where opposite water masses converge, currents might actually be used for rheotactic orientation [53]. During the post-spawning migration out of the Mediterranean Sea, deep-swimming, exhausted tuna would in addition take advantage of the outflowing bottom current to save energy reserves. Other hypothesized causes for deep diving behaviour at this area are predator (namely killer whale) avoidance, and foraging activity [15]. Diet composition analyses reveal in fact that bluefin tuna caught at the Strait of Gibraltar prey on mesopelagic fish and crustaceans (personal observation). Boat noise has been proven to change bluefin school structure and natural swimming direction, inducing abrupt vertical movements towards surface or bottom layers [54]. Hence, the heavy traffic of ships concurring in the narrow passageway between the Atlantic and Mediterranean seas could also be partly responsible for the deep diving behaviour of bluefin tuna at the Strait of Gibraltar.

Distinct changes in the diving behaviour and thermal biology experienced by Atlantic bluefin tuna in the Gulf of Mexico are considered as potential signals of the breeding phase [13]. In agreement with this, the distinctive diving behaviour exhibited in the western Mediterranean regarding HFSD profiles does appear to reflect spawning activity. This diving pattern occurred exclusively in the spawning ground during the putative spawning season, and always became apparent between midnight and dawn, coinciding with the night hours when spawning takes place in the Balearic purse-seine fishing ground [38]. Several individuals tagged in 2011 continued to display this pattern as far as the vicinity of the Alboran Sea, which suggests that the bluefin tuna western spawning area could be broader than generally assumed. This is not surprising, as bluefin tuna show a clear preference to spawn in areas where new and resident Atlantic waters meet generating frontal activity [55], and similar oceanographic structures can occur near the Alboran Sea [56].

Courtship and spawning behaviours were witnessed and filmed during the tagging survey of 2009 (see video S3). Courtship started with grouping of individuals near the sea surface and a few males closely pursuing a female. The fish then took on a darker background colour that enhanced their striped pattern. Eventually, as the spawners released several series of gametes, they shook the caudal fin strenuously to spread and mix them in the water to facilitate fertilisation. Unfortunately, PSAT tags do not allow for internal temperature recordings, but it is plausible that courtship and spawning events, added to the high ambient water temperature in the spawning habitat, cause a significant rise of the internal body temperature that prompts the fish to perform frequent descents below the mixed layer. Repeated brief dives performed by young Pacific bluefin tuna through the thermocline function as an effective behavioural mechanism of thermoregulation in case of both hypothermia and hyperthermia [48]. So, the oscillatory dives starting shortly after the onset of courtship behaviour (from 12:00, UTC time), may play an important thermoregulatory role during spawning of Atlantic bluefin tuna. This hypothesis is consistent with the findings of Teo et al. [13] that during the putative breeding phase in the Gulf of Mexico the fish experience warmer ambient and body temperatures, and may depend to some extent on changes in diving behaviour for thermoregulation.

Along with the thermocline depth and temperature cooling rates, the dissolved oxygen (DO) concentrations could also influence the vertical distribution and diving behaviour of tunas [13,50,57-60]. DO concentrations under 4.3 ml l^-1^ induce decreased heart rate in yellowfin tuna [59,60]. The mixed layer in the Balearic spawning area contains higher DO concentrations (~5 ml l^-1^) [61], whereby the oxygen levels do not appear to be a limiting factor in the spawning environment. However, the metabolic stress caused by courtship-spawning exercises and high ambient temperatures may increase the oxygen requirements [13]. To recover from such energy consuming processes, therefore, the fish could be forced to descend beneath the mixed layer where increased oxygen concentrations (~5.75 ml l^-1^) are encountered [61].

The first diving profile denoting spawning behaviour appeared, on average, 3.3 d following the tag deployment (range: 0-11 d). This low value suggests that the tagging procedure carried out was relatively low stress for the fish. Electronic tag data may help determine the duration of the spawning phase and the spawning frequency, which are important parameters for the assessment of the reproductive potential of tuna stocks [62]. The mean time that the tagged fish spent in the western Mediterranean Sea was 31.2 d; the mean number of spawns estimated from the analysis of diving profiles was 18.3, and the average duration of the spawning phase 23.9 d, which is comparable to that estimated in the Gulf of Mexico [13]. These values, however, may be underestimated, because some fish might have begun to spawn before being tagged. The individuals bearing tags #21 and #25 would provide a good reference regarding the total number of spawns (17 and 25, respectively) and spawning phase extension (24 and 27 d, respectively). These fish did not exhibit spawning behaviour until days 11 and 8 following tagging, respectively, thus their respective tags are likely to have recorded the entire spawning phase. Unlike the estimations of the number of spawns and spawning phase duration, the values of mean spawning frequency (80.3%) and periodicity (1.28 d) obtained from depth and temperature profiles are more reliable as they are based on the relation between spawning and non-spawning days, and hence do not depend on the total number of HFSD profiles recorded by the tags. They are, otherwise, consistent with the spawning periodicity of 1.2 d estimated in previous histological studies based on the postovulatory follicle method [31,33,63].

In conclusion, the use of PSAT tags can contribute to improve our knowledge on Atlantic bluefin tuna life history and spawning behaviour in the East Atlantic and Mediterranean, complementing pre-existing data based on analyses of biological samples. Co-ordinated PSAT tagging surveys spanning the entire Mediterranean Sea would help us to better understand the population structure of the eastern stock and gain a deeper insight into the global population dynamics. Therefore, electronic tagging research should be fostered throughout the full eastern distribution range of the Atlantic bluefin tuna with a view to providing new data for the management and conservation of the species.

## Supporting Information

Video S1 **Underwater PSAT tagging of bluefin tuna in 2009.**
(MP4)

Video S2 **Underwater PSAT tagging of bluefin tuna in 2013.**
(MP4)

Video S3 **Bluefin tuna spawning in the Balearic spawning ground.**
(MP4)
